# The impact of specific pulmonary arterial hypertension therapy on cardiac fluorodeoxyglucose distribution in PET/MRI hybrid imaging–follow-up study

**DOI:** 10.1186/s13550-023-00971-w

**Published:** 2023-03-09

**Authors:** Remigiusz Kazimierczyk, Piotr Szumowski, Stephan G. Nekolla, Lukasz A. Malek, Piotr Blaszczak, Marcin Hladunski, Bozena Sobkowicz, Janusz Mysliwiec, Karol A. Kaminski

**Affiliations:** 1 Department of Cardiology, Medical University of Bialystok, Curie-Sklodowskiej 24a, 15-276 Bialystok, Poland; 2 Department of Nuclear Medicine, Medical University of Bialystok, Curie-Sklodowskiej 24a, 15-276 Bialystok, Poland; 3 Department of Nuclear Medicine, Technical University Munich, Ismaninger Str., 81675 Munich, Germany; 4 Faculty of Rehabilitation, University of Physical Education, Marymoncka 34, 00-968 Warsaw, Poland; 5 Department of Cardiology, Cardinal Wyszynski’ Hospital, Krasnicka Ave 100, 20-718 Lublin, Poland; 6 Department of Population Medicine and Civilization Diseases Prevention, Medical University of Bialystok, Curie-Sklodowskiej 24a, 15-276 Bialystok, Poland

**Keywords:** Pulmonary hypertension, Positron emission tomography, Cardiac magnetic resonance, Prognosis

## Abstract

**Background:**

PET/MRI hybrid imaging in pulmonary arterial hypertension (PAH) provides important prognostic information identifying patients who might benefit from early therapy escalation, as right ventricle (RV) metabolic alterations are linked with hemodynamics and might precede clinical deterioration. Now, we hypothesize that adequate PAH therapy escalation may result in reversal of unfavourable increased glucose uptake of RV, which is associated with improved prognosis.

**Methods:**

Out of twenty-six initially clinically stable PAH patients who had baseline PET/MRI scans, twenty (49.9 ± 14.9 years) had second PET/MRI after 24 months. SUV_RV_/SUV_LV_ ratio was used to estimate and compare cardiac glucose uptake. Occurrences of clinical endpoints (CEP), defined as death or clinical deterioration, were assessed during 48-month follow-up from baseline.

**Results:**

In first 24 months of observation, sixteen patients had CEP and needed PAH therapy escalation. At follow-up visits, we observed significant improvement of RV ejection fraction (45.1 ± 9.6% to 52.4 ± 12.9%, *p* = 0.01), mean pulmonary artery pressure (50.5 ± 18.3 to 42.8 ± 18.6 mmHg, *p* = 0.03), and SUV_RV_/SUV_LV_, which tended to decrease (mean change -0.20 ± 0.74). Patients with baseline SUV_RV_/SUV_LV_ value higher than 0.54 had worse prognosis in 48 months observation (log-rank test, *p* = 0.0007); follow up SUV_RV_/SUV_LV_ > 1 predicted CEP in the following 24 months, regardless of previously escalated treatment.

**Conclusions:**

PAH therapy escalation may influence RV glucose metabolism, what seems to be related with patients’ prognosis. PET/MRI assessment may predict clinical deterioration regardless of previous clinical course, however its clinical significance in PAH requires further studies. Importantly, even mild alterations of RV glucose metabolism predict clinical deterioration in long follow-up.

*Clinical Trial Registration* ClinicalTrials.gov, NCT03688698, 05/01/2016, https://clinicaltrials.gov/ct2/show/study/NCT03688698?term=NCT03688698&draw=2&rank=1

## Background

Progressive increase of right ventricular (RV) afterload leading to RV failure is inevitable in poorly managed or undiagnosed pulmonary arterial hypertension (PAH) [1, 2]. Despite the availability of multiple prognostic parameters, it is still a challenge to describe RV function properly, especially prior to first clinical deterioration [3, 4]. Early detection of PAH progression with a use of newly discovered prognostic parameters could help to identify group of patients requiring rapid therapy escalation or lung transplantation and more over avoid potential wrong therapeutical decisions resulting in patient’s death [5–7]. In our previous studies, we presented novel approach for PAH diagnostics [5, 8, 9]. Simultaneous positron emission tomography/magnetic resonance (PET/MRI) hybrid imaging plays an emerging role in PAH combining accurate assessment of RV’s anatomy and function with its metabolic alterations. We confirmed on a group of stable PAH patients that PET/MRI could be a useful tool in patients’ management providing not only a better insight into hemodynamics but also important prognostic information. Our results proved that changes in glucose metabolism in cardiomyocytes, presented as 18^F^-flurodeoxyglucose (18-FDG) uptake in PET are linked with hemodynamic deterioration (obtained by MRI or right heart catheterization (RHC)), preceding clinical worsening. Patients with higher standardized glucose uptake (SUV) of RV than left ventricle (presented as parameter SUV_RV_/SUV_LV_ ratio) had worse prognosis especially when combined with deteriorated RV ejection fraction (RVEF) obtained from MRI [5]. Proposed PET/MRI parameters may be especially helpful in identification of a higher risk group in apparently stable PAH patients. Currently, patients’ assessment is based on widely available and often used risk parameters like 6-min walking test distance (6MWT), cardiac index (CI) or brain natriuretic peptide (BNP) levels, which mostly failed to precisely predict prognosis in the groups of medium risk patients [1, 10, 11]. Use of PET/MRI in this particular group of patients may result in better management and improved prognosis. Right therapeutic decisions may also bring economic benefits – patients may be treated more effectively with properly optimized therapy.

Most of the previous studies attributed “metabolic shift” occurring in cardiomyocytes to pressure overload, focused on its relationship with other PAH parameters e.g. RV ejection fraction (RVEF), mean pulmonary artery pressure (mPAP) or pulmonary vascular resistance (PVR), but it may also be associated with increased inflammatory signalling [12, 13]. As half of enrolled PAH patients in our previous study presented significantly unfavourable SUV_RV_/SUV_LV_ ratio > 1, it seems that lowering this parameter below 1 should positively affect their prognosis [5]. Therefore, we decided to check whether the abnormal SUV_RV_/SUV_LV_ can be reversed with the help of appropriately selected specific PAH treatment. In all PAH patients with baseline SUV_RV_/SUV_LV_ ratio > 1 and/or RVEF < 40% we escalated PAH therapy within one year (as they eventually experienced clinical end-point) and performed second PET/MRI scans exactly after 24 months since baseline scans. Additional complex assessments of PAH patients helped us to verify previously described relationship between PET/MRI and RHC parameters and to determine whether PET/MRI imaging should be routinely performed in PAH.

## Methods

### General overview of the study

**Fig. 1:**
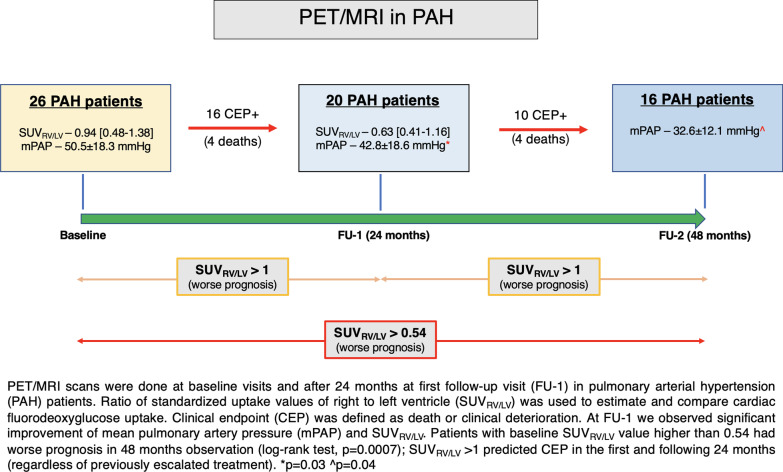
Graphical presentation of the study

**Fig. 2:**
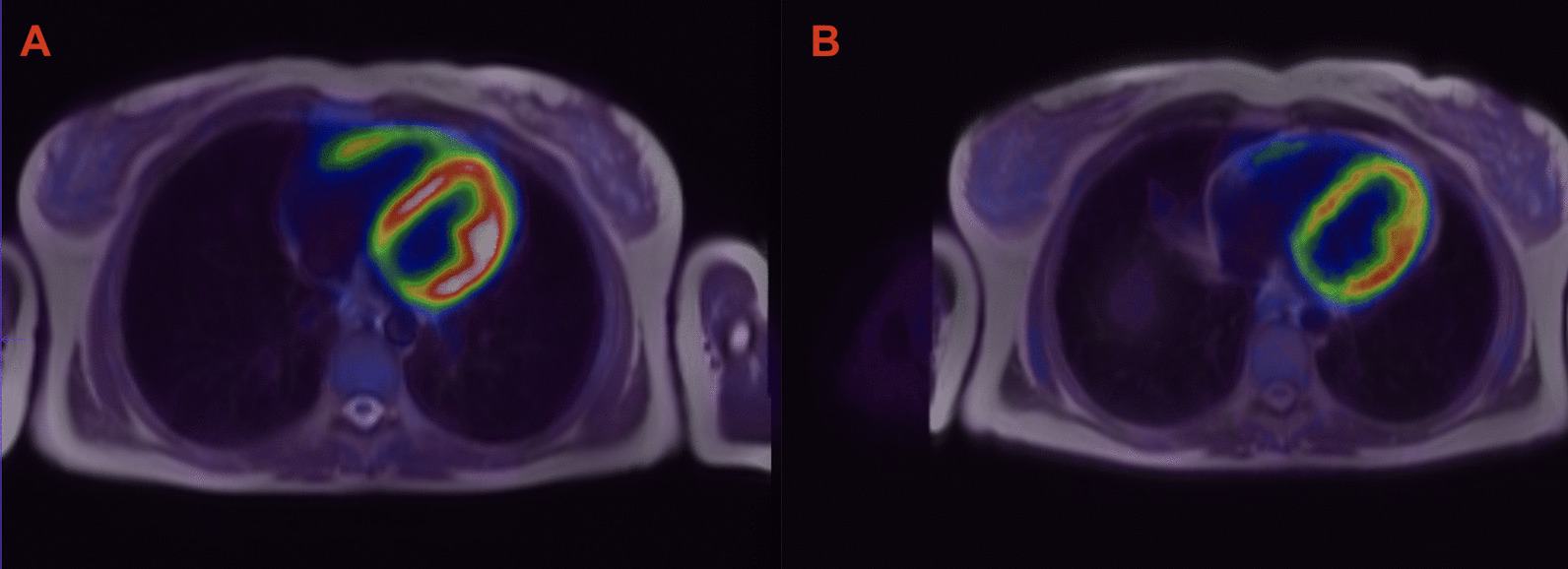
Cardiac 18-fluorodeoxyglucose uptake in pulmonary hypertension patient before **A** initiation of specific therapy and after **B** 24 months of treatment

Baseline visit details (study and control groups characterizations, inclusion/exclusion criteria of the study) are presented in our previous article [5]. Patients were clinically followed-up on regular three-month basis, however the first structured (including PET-MRI, echo, 6MWT, biochemical analysis) follow-up visit (FU-1) was done after 24 months from the baseline visits (graphical presentation of the study Fig. 1). PET/MRI scans were done together with other tests during the same hospitalization, in the same patients’ hemodynamic state (Fig. 2). Occurrence of clinical end-points (CEP) was checked between baseline visits and FU-1 visits. CEP was defined as death or WHO class worsening and/or with hospitalisation due to pulmonary hypertension progression or right heart failure (as described previously) [5]. All CEP( +) patients eventually had PAH therapy escalation within 1 month since CEP. After FU-1 visits we observed study group for next 24 months for occurrence of CEP (defined the same as above), ending in second follow-up visit (FU-2). PET/MRI scans were not repeated after FU-1 visit (during FU-2 only WHO class, laboratory tests, 6MWT and RHC were done). The clinical follow-up lasted in total 48 months.

Therapy escalation was defined as initiation of prostacyclin (PGI) treatment (parenteral or oral) or adding second-line oral therapy according to ESC Guidelines [1]. Right heart catheterization was repeated at FU-1 and FU-2 visits in standard technique within median 6 [2–9] days of PET/MRI scans with a use of previously described protocol [5].

### Myocardial PET/MRI hybrid imaging

PET/MRI imaging was performed with a 3 T Biograph mMR hybrid system (Siemens, Healthcare Erlangen, Germany) at the time of enrolment and at FU-1 visits. Myocardial PET imaging and MRI analysis were performed as previously described [5, 14, 15]. 18-FDG uptake is presented as a ratio of standardized uptake value of RV to LV (SUV_RV_/SUV_LV_).

### Statistical analysis

The data are presented as a mean ± standard deviation (SD) or median [interquartile range] as appropriate. Statistical analysis was performed using Student’s t-test or Mann–Whitney U test for continuous data depending on distribution and Wilcoxon signed-rank test to compare repeated parameters in individual cases. Spearman’s correlation coefficient was used to examine the relationship between two continuous variables. Benjamini–Hochberg correction was used to account for multiple comparisons in correlation analysis. Receiver operator characteristic curves (ROC) were plotted to determine the area under the curve (AUC) and sensitivity and specificity of the optimal cut-offs (binomial method). DeLong’s test was used to compare two AUC results. To investigate the occurrence of clinical endpoints Kaplan Meier method with log-rank test was implemented. *p* < 0.05 was deemed statistically significant. A statistical software package STATA V.13 (USA) was used for the analysis.

## Results

### Group characteristics

The general characteristics of study group (*n* = 26) at baseline visit (and comparison with control healthy group) are presented in previous manuscript [5]. PAH group median SUV_RV_/SUV_LV_ ratio was 1.02 [0.42–1.21] and control group was 0.16 [0.13–0.25], *p* < 0.001. At baseline visit patients with PAH had low BNP level (78.8 pg/ml [46–282]) and mean distance in 6-min walking test was 382 ± 102.3 m. Most of the enrolled patients were in WHO Class III and mPAP was 48.9 ± 18.7 mmHg.

**Fig. 3:**
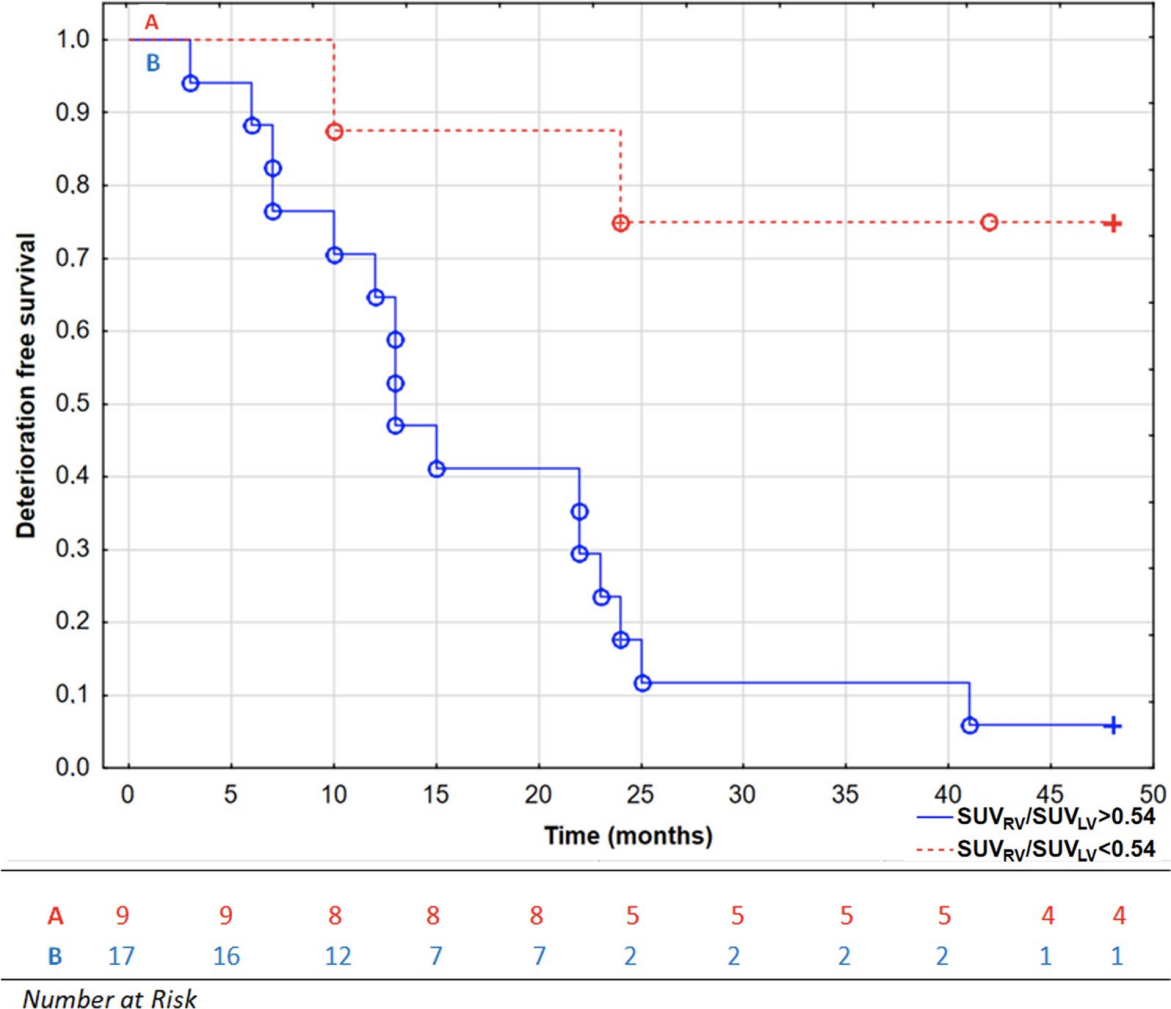
Kaplan–Meier curves presenting deterioration-free survival in patients with pulmonary arterial hypertension based on SUV_RV_/SUV_LV_ ratio in 48 months of observation, log-rank test, *p* = 0.0007. °, complete events; + , censored events; *LV* Left ventricle, *RV* Right ventricle, *SUV* Standardized uptake value

In current study, we observed PAH group for 48 months and verified previously published results considering prognostic role of SUV_RV_/SUV_LV_. In total, 19 patients (73%) had experienced CEP. Mean time to clinical worsening was 25.2 ± 16.1 months. ROC analysis revealed that in extended observation cut-off value of SUV_RV_/SUV_LV_ parameter was 0.54 (AUC: 0.86 (0.69–0.98), *p* < 0.001), suggesting that even subtle shift in cardiac glucose metabolism in PAH may result in worse prognosis (median SUV_RV_/SUV_LV_ in control healthy group was 0.16 [0.13–0.25]). PAH patients with SUV_RV_/SUV_LV_ higher than 0.54 had significantly worse prognosis in long term (log-rank test, *p* = 0.0007, Fig. 3).

There were eight deaths in total, 30% of baseline number of patients – all of them were related with rapid PAH progression. Analysis of small subgroup of PAH patients who died revealed that they had significantly higher WHO class at baseline visit (2.75 ± 0.46 vs 2.0 ± 0.50, *p* = 0.01), BNP concentrations (456 ± 280 vs 164 ± 47 pg/ml, *p* = 0.006) and lower 6MWT distances (312 ± 90 vs 425 ± 83 m, *p* = 0.01). No other significant changes in hemodynamic or imaging parameters were observed. However, seven patients who died (88%) had either a SUV_RV_/SUV_LV_ ratio > 1 or a RVEF < 40% at baseline, what confirms our previous results about the prognostic role of PET/MRI patients’ assessments [5].

### First follow-up visits

After 24 months from baseline visits, we performed first structured follow-up (FU-1) visits. Twenty PAH patients remained in the study group (4 deaths, 2 patients did not agree to participate in the follow-up visits). Patients’ characteristics and the comparison of functional, PET/MRI/RHC parameters obtained at all three visits (baseline, FU-1, and FU-2) are presented in Table 1. At FU-1, we observed significant change of MRI-derived RVEF (45.1 ± 9.6 to 52.4 ± 12.9%, *p* = 0.01), and improvement in hemodynamic parameters obtained from RHC e.g., mPAP (50.5 ± 18.3 to 42.8 ± 18.6 mmHg, *p* = 0.03) and PVR (8.9 ± 5.7 to 7.15 ± 4.2WU, *p* = 0.04). Mean change of SUV_RV_/SUV_LV_ (follow-up scans to baseline) was − 0.20 ± 0.74 confirming that most of the patients had decreased glucose metabolism in RV cardiomyocytes. Importantly, there was no significant change in RV mass/BSA parameter. Median FDG uptake of this group, presented as SUV_RV_/SUV_LV_ tended to decrease from 0.94 [0.48–1.38] to 0.62 [0.41–1.16], *p* = 0.19, but 7 patients (35%) still had SUV_RV_/SUV_LV_ ratio higher than 1. Interestingly, patients who had improvement in SUV_RV_/SUV_LV_ (lower follow-up value than baseline, *n* = 12) had significantly higher baseline mPAP (56 ± 20.5 vs 42.4 ± 10.9 mmHg, *p* = 0.04) but improved cardiac index at FU-1 (from 2.47 ± 0.45 l/min/m^2^ to 2.98 ± 0.39, *p* = 0.007). Follow-up SUV_RV_/SUV_LV_ ratio significantly correlated with follow-up RV hemodynamic parameters confirming strong relationship between RV function and metabolic alterations (Table 2).

**Table 1:** Basic characteristics of pulmonary arterial hypertension (PAH) group at baseline and two follow-up visits (after 24 and 48 months)

	Baseline	FU-1	FU-2
Subjects, *n*	20*	20	16
CEP (deaths)		16 (4)	10 (4)
Age, years	49.2 ± 15.1	49.8 ± 14.9	50.1 ± 14.4
Sex (females), % (*n*)	70 (14)	70 (14)	81 (13)
WHO Class	2.1 ± 0.7	2.3 ± 0.7	2.2 ± 0.7
6 min walking test distance,* m*	404 ± 87.8	412 ± 77	420 ± 111
BNP, pg/ml	90.8 [46–282]	114 [77–245]	113 [76–252]
*PAH etiology*			
Idiopathic/heritable PAH, % (*n*)	60 (12)	60 (12)	56 (9)
Connective tissue disease related PAH, % (*n*)	15 (3)	15 (3)	12 (2)
Congenital heart disease related PAH, % (*n*)	25 (5)	25 (5)	32 (5)
*PAH specific therapy*			
Phosphodiesterase type 5 inhibitors, % (*n*)	38 (10)	10 (2)	12 (2)
Endothelin receptor antagonists, % (*n*)	11 (3)	15 (3)	0 (0)
Prostacyclins, % (*n*)	20 (5)	65 (13)	50 (8)
Phosphodiesteraze type 5 inhibitors + endothelin receptor antagonists, % (*n*)	31 (8)	10 (2)	38 (6)
*Hemodynamics*			
Systolic pulmonary artery pressure, mm Hg	82.2 ± 29.2	78.2 ± 24.2	75.3 ± 32.3
Diastolic pulmonary artery pressure, mm Hg	33.8 ± 14.3	31.2 ± 13.9	29.43 ± 14
Mean pulmonary artery pressure, mm Hg	50.5 ± 18.3	42.8 ± 18.6^	32.6 ± 12.1^^
Pulmonary capillary wedge pressure, mm Hg	10.6 ± 2.5	9.73 ± 3	8.8 ± 2.8
Pulmonary vascular resistance, Wood units	8.9 ± 5.7	7.3 ± 4.7^	5.3 ± 2.8^^
Cardiac index, L/min/m^2^	2.5 ± 0.4	2.9 ± 0.4^	2.9 ± 0.6^^
Right atrium pressure, mm Hg	8.6 ± 3.6	8.1 ± 5.3	8 ± 5.3
*RV parameters (MRI)*			
RV ejection fraction, %	45.1 ± 9.6	52.4 ± 12.9^	
RV EDV/BSA, mL/m^2^	113.2 ± 24.5	106 ± 27	
RV ESV/BSA, mL/m^2^	62.7 ± 22.7	50 ± 11	
RV mass/BSA, g/m^2^	39.9 ± 13.9	39.2 ± 14.6	
RV compacted myocardium thickness, mm	5.7 ± 1.5	5.2 ± 1.3	
Pulmonary arterial compliance, mL/mm Hg	2.4 ± 1.8	3.2 ± 2.4^	
Right ventricle stroke work index, g*m*m^2^/beat	20.6 ± 8.4	18.2 ± 7.5	
*Myocardial metabolism (PET)*			
SUV_RV_	2.5 [1.4–5.5]	3.92 [1.6–8.1]	
SUV_LV_	3.5 [2.1–6.6]	5.7 [4.8–8.9]	
SUV_RV_/SUV_LV_ ratio	0.94 [0.48–1.38]	0.63 [0.41–1.16]	

Data presented as mean ± SD (normal distribution; paired t-test was used to compare two variables) or median [IQR] (non-normal distribution; Wilcoxon signed rank test was used to compare two variables)

^*^number of matched pairs of patients present at both baseline and FU-1 visits. ^ statistical significance (*p* < 0.05) (Wilcoxon signed rank test was used to compare matched (Baseline vs FU-1) values). ^^ statistical significance (*p* < 0.05) (Wilcoxon signed rank test was used to compare matched (Baseline vs FU-2) values)

*BNP* B-type natriuretic peptide, *BSA* Body surface area, *CEP* Clinical end-point, *EDV* End-diastolic volume, *ESV* End-systolic volume, *FU* Follow-up, *LV* Left ventricle, *MRI* Magnetic resonance imaging, *PET* Positron emission tomography, *RV* Right ventricle, *SUV* Standardized uptake value, *WHO* World Health Organisation

**Table 2:** Spearman’s correlations between follow-up PET-derived and MRI/RHC-derived parameters

Correlation coefficient (*r*); *p*-value	SUV_RV_/SUV_LV_
6MWT	*r* = − 0.49; *p* = 0.02
BNP	*r* = 0.58; *p* = 0.007*
RV thickness	*r* = 0.41; *p* = 0.04*
RVEF	*r* = − 0.60; *p* = 0.004*
mPAP	*r* = 0.79; *p* = 0.00002*
PVR	*r* = 0.82; *p* = 0.000008*
PAC	*r* = − 0.73; *p* = 0.0003*
RVSWI	*r* = 0.77; *p* = 0.0001*

^*^ *p*-value significant (< 0.05) after Benjamini–Hochberg correction

*BNP* B-type natriuretic peptide, *FU* Follow-up, *LV* Left ventricle, *MRI* Magnetic resonance imaging, *mPAP* Mean pulmonary arterial hypertension, *6MWT* 6 min walk test distance, *PAC* Pulmonary arterial compliance, *PET* Positron emission tomography, *PVR* Pulmonary vascular resistance, *RHC* Right heart catheterization, *RV* Right ventricle, *RVEF* Right ventricle ejection fraction, *RVSWI* Right ventricle stroke work index, *SUV* Standardized uptake value

Furthermore, at FU-1 visit it occurred that 16 patients (61%) had experienced clinical end-point during 24-months follow-up (4 deaths, 12 had clinical symptoms of PAH progression). CEP patients presented lower 6MWT distance (*p* = 0.01), higher SUV_RV_/SUV_LV_ ratio (*p* = 0.005) and mPAP (*p* = 0.0002) and lower RVEF (*p* = 0.002), what is consistent with our previous results [5].

All CEP + patients had PAH therapy escalation between baseline and FU-1 visits—twelve patients started parenteral PGI (treprostinil or epoprostenol), one patient oral PGI analogue (treprostinil), two patients had added second-line drug – macitentan and one – inhaled iloprost. Five patients, who eventually died in that time, had PGI treatment initiated during hospitalizations preceding death, thus only eight patients on PGI therapy continued the study.

### Second follow-up visits

We observed the study group for another 24 months (48 months in total since baseline visit). Between FU-1 and FU-2, 10 patients had CEP (three patients for the first time in the study) including four deaths.

**Fig. 4:**
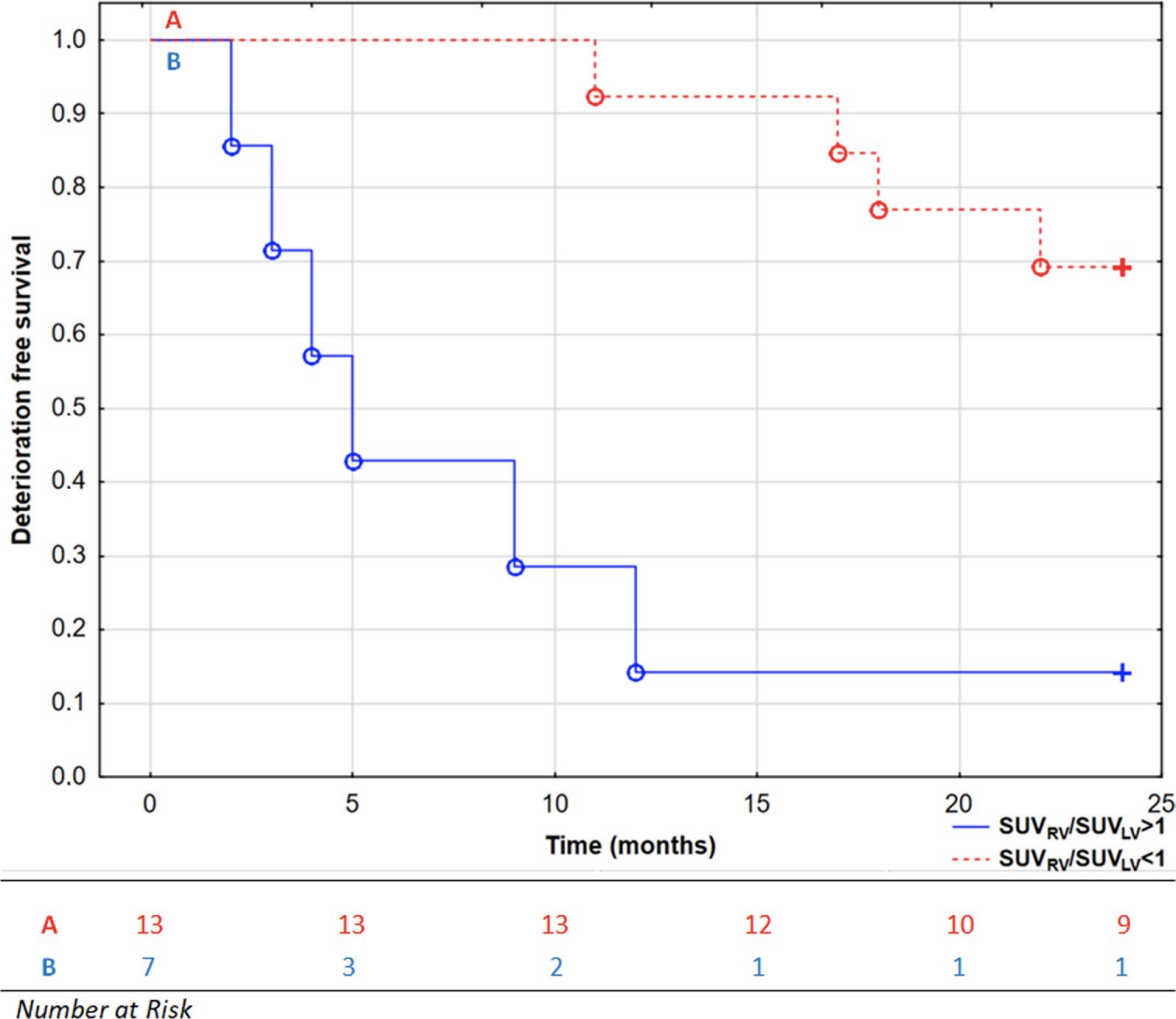
Kaplan–Meier curves presenting deterioration-free survival in patients with pulmonary arterial hypertension based on SUV_RV_/SUV_LV_ ratio after follow-up PET/MRI scans, log-rank test, *p* = 0.006. °, complete events; + , censored events; *LV* Left ventricle, *RV* Right ventricle, *SUV* Standardized uptake value

It occurred that second assessments of SUV_RV_/SUV_LV_ ratio and RVEF done at FU-1 visits had also prognostic significance. ROC analysis revealed that both SUV_RV_/SUV_LV_ and RVEF had similar prediction for second CEP (AUC: 0.8 (0.60–0.96) vs 0.93 (0.81–0.97), *p* = 0.24, respectively). Patients with second SUV_RV_/SUV_LV_ > 1 had significantly worse prognosis after FU-1 visits (log-rank test, *p* = 0.006, Fig. 4).

At FU-2 visits a significant improvement we observed only in RHC parameters (mPAP, PVR, CI) in the group of sixteen patients who were present at FU-2 (Table 1).

### Impact of specific PAH therapy on PET results

Out of twelve PAH patients who had improvement in SUV_RV_/SUV_LV_ ratio (mean improvement − 0.59 ± 0.39; 33.86 ± 25% of baseline value) between baseline and FU-1 visits, eight had PAH therapy escalation to PGI (initiation of parenteral PGI in seven patients, oral PGI in one patient). Subgroup of patients who eventually received PGI treatment had baseline higher SUV_RV_/SUV_LV_ ratio (1.09 [0.78–2.1] vs 0.52 [0.29–1.32], *p* = 0.03), mPAP (58.42 ± 15.96 vs 42.3 ± 15.5 mmHg, *p* = 0.01), and lower RVEF (37.82 ± 9.05 vs 48.82 ± 9.11%, *p* = 0.009).

## Discussion

The prognostic significance of FDG uptake ratio between the RV and LV for PAH patients’ prognosis was previously described [5, 6]. However, this study shows for the first time that adequate PAH targeted therapy may reverse unfavourable glucose metabolic alterations in RV myocytes and that even single SUV_RV_/SUV_LV_ ratio measurement could be an additional prognostic tool in the short- and long-term.

We previously showed that subtle changes in myocardium metabolism (obtained by non-invasive PET) are in strong association with hemodynamic impairment which is often clinically silent. Published results were verified in this study as almost all patients with higher RV glucose uptake than LV (SUV_RV_/SUV_LV_ ratio > 1) experienced combined clinical end-point including deaths in just 24 months. Now, the prognostic significance of SUV_RV_/SUV_LV_ > 1 was also confirmed in the time between FU-1 and FU-2 visits (next 24 months) in the group of prevalent, previously treated PAH patients. This suggests that SUV_RV_/SUV_LV_ ratio value higher than 1 may be universal, helpful tool to estimate PAH patient prognosis not only at diagnosis but also after initiation and/or escalation of PAH specific treatment.

PET/MRI allows to detect the RV dysfunction and/or metabolic alterations before clinical deterioration and theoretically to start/escalate treatment early enough. Perhaps, PAH therapy escalation to advanced agents e.g. parenteral PGI (treprostinil, epoprostenol, iloprost) requires (according to current ESC Guidelines[1]) the lack of improvement in objective and verified parameters e.g. CI or 6MWT. Thus, we could not change the treatment based only on unfavourable PET/MRI results. In our study, all patients who received more advanced treatment (including PGI) have already experienced clinical end-point and did not meet improvement criteria at hospitalizations which took place between baseline and FU-1 visits. Importantly, all patients who eventually received PGI treatment had not only worse hemodynamic parameters (mPAP, RVEF) at baseline, but also SUV_RV_/SUV_LV_ ratio was higher. This indicates potential benefits of baseline PET imaging in PAH patients.

Follow-up PET/MRI scans together with comprehensive assessments allowed us to compare changes of newly proposed parameters (SUV_RV_/SUV_LV_) with established prognostic factors (WHO class, 6MWT distance, RVEF or mPAP). Despite of significant improvement of mean mPAP and RVEF values at FU-1 visits, no statistically significant decrease of median SUV_RV_/SUV_LV_ ratio was observed study group. However, in patients who had improvement in this ratio (lower follow-up value than baseline, *n* = 12), we observed significant increase of established PAH prognostic factor—cardiac index at FU-1. This suggests that RV FDG accumulation in advanced PAH may decrease after the specific PAH-treatment in accordance with the degree of reduction in the pulmonary pressure/resistance, leading to increase of cardiac index.

We are aware, that group of 20 patients is too small to objectively indicate the possible place for repeating PET/MRI scans in PAH patients. Only Oikawa et al. confirmed before (in the group of 10 PAH patients treated with epoprostenol) that the change of the SUV_RV_ was significantly correlated with the percentage change of the PVR [16].

Our results only suggest that repeating this imaging method may help to determine not only the effectiveness of therapy but also further prognosis. During extended observation after FU-1 visits, we have documented additional clinical deteriorations. Despite of adequate treatment and slight reduction of RV FDG uptake observed in PET, patients with still extremely elevated SUV_RV_/SUV_LV_ > 1 had worse prognosis. Thus, depicted subgroup of patients with advanced PAH (e.g. based on unfavourable PET/MRI/RHC parameters) could be often monitored and receive aggressive targeted treatment (together with accelerated qualification for lung transplantation).

Importantly, our extended follow-up observation up to 48 months revealed, that PAH patients with just mildly increased (comparing to physiological) SUV_RV_/SUV_LV_ ratio > 0.54 had already worse prognosis. Most previous studies, including ours, based on shorter observation showed that only severely increased RV FDG uptake (like SUV_RV_/SUV_LV_ > 1) affects patients’ prognosis [5, 6]. Now, we present that PAH patients with baseline SUV_RV_/SUV_LV_ slightly higher – 0.54 than physiological range may experience clinical deterioration in long-term observation despite of followed initiation of specific treatment. It seems that any increase of glucose metabolism in RV in PAH rather than observed in healthy subjects may result in progressive PAH development. This confirms the possible important long term prognostic information from FDG uptake assessment (together with PAH established parameters).

As we still are not sure whether observed metabolic shift in cardiomyocytes is secondary to or it is a primer cause of RV impairment, presented findings emerge the possible need of potential new therapeutic targets affecting cardiac metabolism [17, 18]. Statistically significant correlations between PET/MRI parameters and hemodynamic measurements observed both at FU-1 and FU-2 visits suggest that there is a strong relationship between affected pulmonary arteries and RV cardiomyocytes. Since modulation of pulmonary vascular processes (with a use of PAH specific treatment) is associated with improvement of right ventricular hemodynamics and thus patients’ prognosis, it seems that possible modulation of right ventricular metabolism may have a similar effect. The study underlines emerging role of further research in the field of possible metabolic modulations therapies in PAH.

We are aware that PET/MRI hybrid imaging system is still expensive and not widely available diagnostic method. Thus, it will be probably limited to rare cases. However, this study shows that even single PET analysis (or PET/CT) is helpful to obtain important additional information about PAH advancement. On the other side, possible MRI assessment (especially performed simultaneously) provides many other newly proposed parameters like RV global longitudinal strain or late gadolinium enhancement mass index, which in direct comparisons (done in other pilot studies) have better prognostic significance in PAH than established ones [8, 9].

Limitations of the study is relatively small study group, which is still equivalent to similar research in this field. Unfortunately, most of the initially enrolled patients were already receiving oral specific treatment, thus we could only check the effectiveness of PAH therapy escalation in second or third line. As prostacyclins are considered the most effective PAH drugs[1], we thought that their initiation should be effective enough to act on possible glucose cardiac uptake change.

It should be underlined that the objective role of PET/MRI parameters in PAH patients requires larger prospective study (on bigger group of naïve patients) with therapeutic decisions based on periodic SUV_RV_/SUV_LV_ assessments.

## Conclusions

To summarize, subtle cardiac metabolic changes observed in PET/MRI imaging in PAH patients are related with RV dysfunction and may help to determine effectiveness of targeted therapy and prognosis. Early assessment of possible RV glucose uptake alteration seems to be helpful in PAH patients’ management in the short and long term.

## Data Availability

The datasets used and/or analyzed during the current study are available from the corresponding author on reasonable request.

## Abbreviations

18F-FDG: 18F-fluoro-2-deoxyglucose
BSA: Body surface area
CO: Cardiac output
CT: Computed tomography
mPAP: Mean pulmonary artery pressure
MRI: Magnetic resonance imaging
PAH: Pulmonary arterial hypertension
PAWP: Pulmonary artery wedge pressure
PET: Positron emission tomography
PH: Pulmonary hypertension
PVR: Pulmonary vascular resistance
RHC: Right heart catheterization
ROC: Receiver operator characteristic
RV: Right ventricle
RVEF: Right ventricle ejection fraction
SUV: Standard uptake value
SV: Stroke volume
WHO: World Health Organization
WU: Wood’s units
